# A Generic Program for Multistate Protein Design

**DOI:** 10.1371/journal.pone.0020937

**Published:** 2011-07-06

**Authors:** Andrew Leaver-Fay, Ron Jacak, P. Benjamin Stranges, Brian Kuhlman

**Affiliations:** Deptartment of Biochemistry, University of North Carolina, Chapel Hill, North Carolina, United States of America; University of South Florida College of Medicine, United States of America

## Abstract

Some protein design tasks cannot be modeled by the traditional single state design strategy of finding a sequence that is optimal for a single fixed backbone. Such cases require multistate design, where a single sequence is threaded onto multiple backbones (states) and evaluated for its strengths and weaknesses on each backbone. For example, to design a protein that can switch between two specific conformations, it is necessary to to find a sequence that is compatible with both backbone conformations. We present in this paper a generic implementation of multistate design that is suited for a wide range of protein design tasks and demonstrate *in silico* its capabilities at two design tasks: one of redesigning an obligate homodimer into an obligate heterodimer such that the new monomers would not homodimerize, and one of redesigning a promiscuous interface to bind to only a single partner and to no longer bind the rest of its partners. Both tasks contained *negative design* in that multistate design was asked to find sequences that would produce high energies for several of the states being modeled. Success at negative design was assessed by computationally redocking the undesired protein-pair interactions; we found that multistate design's accuracy improved as the diversity of conformations for the undesired protein-pair interactions increased. The paper concludes with a discussion of the pitfalls of negative design, which has proven considerably more challenging than positive design.

## Introduction

The last fifteen years have produced remarkable advances in the field of protein design [1]–[3], with the redesign and stabilization of existing proteins [4]–[10], the *de novo* design of protein structures [11], [12], the altering of existing protein functionality [13], [14], the redesign of existing protein/protein interfaces [15], [16], and the design of new enzymes [17]–[19]. The advances have come primarily from the use of computational approaches: the challenge of choosing a sequence to perform a desired task is formulated as an optimization problem which can be given to a computer to solve. Typically, the backbone of a particular protein is held fixed and the conformations of its sidechains (and their amino acid identities) are altered to minimize an energy function. The conformations of the side chains are taken from observed conformations from the Protein Data Bank (PDB) called “rotamers” [20]–[22] and are typically represented with all their atoms including hydrogens. The energy functions being optimized are often built from those available in molecular dynamics packages and include terms for van der Waals interactions, hydrogen bonding, solvation, electrostatics, and torsional strain. This standard formulation of minimizing the energy of a sequence on a fixed backbone has proven very useful in a variety of tasks: in *de novo* design, finding a low energy sequence compatible with a given backbone has been used to produce several proteins that adopt that backbone conformation; in protein interface design, finding a low-energy sequence compatible with a particular docked orientation of the two proteins has been used to produce an interaction between the two proteins [23].

Not all protein design tasks can be modeled by optimizing the sequence for a single structure. For example, one might want to redesign the homodimeric interface of Immunoglobulin G, IgG, (as Genentech has [24]) to create heterodimeric antibodies that, in turn, can be loaded with two variable domains specific for two different substrates. Indeed, such “bispecific antibodies” are increasingly common in the treatment of cancer and show promise in other therapies [25]. Redesigning the homodimeric interface of IgG requires coming up with a sequence that not only allows for the two monomers to heterodimerize but also ensures that neither of the monomers homodimerize. With the standard protein design formulation, the optimization of the sequence for a single structure (in this case, the heterodimer) leaves the design algorithm blind to whether or not the monomers could homodimerize. The problem stems from the fact that the standard design methodology can only examine a single state of the protein at one time; the solution to this problem is simply to model multiple states simultaneously.

In multistate design, the optimization problem is not as straight forward as in single state design; instead of optimizing the energy for one state, one has to find a sequence that has a good energy for one state and possibly a bad energy for another. To this end, multistate design requires a “fitness function” to rank sequences based on how well they meet the goals of a particular design task. The fitness function is evaluated by first threading a single sequence onto multiple states, calculating the energy of that sequence on each state, and finally combining those energies to produce a single value. A significant hurdle in solving a multistate design problem is formulating a fitness function that captures the design goals. Havranek and Harbury, in trying to design homodimeric coiled coils that would not also heterodimerize, maximized the probability of homodimer formation by using a partition function that included the energies of competing heterodimeric states and an aggregate state [26]. Ambroggio and Kuhlman optimized the sum of the energies for two conformations of a single sequence so that it would form a monomer in the presence of zinc, and a trimer in its absence [27]. Grigoryan, Reinke, and Keating optimized the energy of Bzip-peptide heterodimerization under the constraint that the energy gap between heterodimers and homodimers exceed some threshold [28]. Ashworth *et al.* optimized the specificity of the I-Msol1 homoendonuclease by favoring the binding energy for I-Msol1 to the target DNA sequence over alternate DNA sequences [29].

This paper presents a generic multistate design implementation for solving arbitrary multistate design problems: the software is generic in that it allows the user to program their fitness function from a text file, encouraging the user to search through fitness-function space, and not just sequence space. We test our implementation at two design tasks: a *heterodimerization task* wherein an existing homodimer is redesigned so that the new monomers heterodimerize, but do not homodimerize, and an *orthogonal interface redesign task* wherein a promiscuous protein A, which naturally binds proteins B, C and D, is redesigned so that A continues to bind B, but no longer binds C or D. We demonstrate, *in silico*, the success of our mulitistate design program at each of these tasks. In the heterodimerization task, we show that the heterodimeric species is favored over the homodimeric species, and that multistate design does a better job than single-state design in disfavoring the homodimers. In the orthogonal interface redesign task, we show that we can preserve the AB binding energy, while substantially decreasing the AC and AD binding energies.

Both design tasks feature *negative design* in that there are interactions between certain pairs of proteins which must be destabilized to meet the design goals. This can be captured in the fitness function by subracting the energies for the states reprefosenting the undesired interactions (called *negative states*) from the energies of the desired interactions (called *positive states*). Typically, multistate design destabilizes the negative states by introducing collisions across the interface; however, these collisions are often easily resolved by separating the proteins slightly. In such a case, multistate design would predict that it has destabilized the interaction for a pair of proteins, yet subsequent redocking can find low-energy conformations for them. If multistate design only considers a single conformation for each negative state, then its predictions for their energies contain substantial amounts of error. We solve this problem by allowing the negative states to choose the lowest energy conformation among a large set of available conformations, including those partially separated conformations. We generate this set of conformations in an iterative fashion by redocking the outputs generated by multistate design. In each “round,” we perform multstate design and follow it with docking of the negative states. If the energies of the negative states predicted by multistate design greatly disagree with the energies produced by docking, we continue on to the next round, expanding the set of conformations for the negative states. We demonstrate *in silico* what had previously been hypothesized about this approach [26]: that representing many conformations for the negative states improves the accuracy of multistate design.

In keeping with the theme of this special collection, we also include a full description of how to repeat our computational experiments as a “protocol capture” included in the Supporting Information File S1. This includes an in-depth description of the input-file formats, as well as the set of input files, command lines, and job-control scripts used in this study.

## Methods

### Software

Our software separates its search through sequence space and its search through side chain conformation space. A genetic algorithm explores sequence space in an outer loop, and each state optimizes its rotamers for a given sequence in an inner loop. The energies produced in this inner loop are fed to a user-defined fitness function that guides the genetic algorithm's search through sequence space. To keep simulations fast, the implementation uses MPI to distribute the inner-loop calculations across multiple processors. The software is written as part of the Rosetta3 molecular modeling suite [30] and will be available in the 3.3 release. We rely on Rosetta's standard “score12” score function [31] and refer to units of this score function when referring to Rosetta Energy Units (REU).

#### Genetic Algorithm

The genetic algorithm, described first in the context of mulitistate design by Havranek and Harbury [26] and whose implementation comes from Ashworth *et al.* [29], maintains a population of 100 sequences and is run for 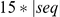 generations, where 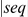 is the length of the sequence being designed (i.e. the number of positions being mutated). Between generation 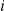 and generation 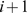, the genetic algorithm propagates the 50 sequences with the best (lowest) fitness, and generates 50 new sequences with 98% generated as point mutants from the best 50 sequences of the previous generation, and 2% generated as crossover combinations of existing sequences. These parameters were chosen by testing the algorithm at interface sequence recovery with a fitness function described by the energy of the complex – effectively, single-state design. These parameters yielded energies and sequences similar to Rosetta's existing single-state design algorithm.

#### State Definition

A state in our implementation refers to one of the many possible structures on which a sequence is being optimized. Each state is defined by three things: 1) a fixed backbone scaffold, 2) a mapping between some or all of the residues on this scaffold and positions in the sequence being optimized in the outer loop, and 3) a secondary rotamer-optimization file. The fixed backbone scaffold is given by a PDB file. The mapping is given in a *correspondence file* that lists which residues on the scaffold take their identify from which positions in the sequence optimized by the genetic algorithm (e.g. “residue 24 on chain A takes its identity from position 3 in the sequence the genetic algorithm optimizes”). The rotamers for each of the residues listed in the correspondence file are optimized in each iteration through the outer-loop. The secondary rotamer-optimization file, called a *secondary resfile* defines which residues in addition to those listed in the correspondence file should also have their rotamers optimized.

#### Rotamer Optimization

At the start of execution, the program builds a fixed set of rotamers for all allowed amino acids at each residue for each state. When a particular sequence is assigned to a state, the program selects the appropriate subset of rotamers and performs rotamer optimization with this subset. It uses a slight variation on the original FASTER algorithm [32] of first assigning the backbone-minimum-energy conformation (BMEC) and then performing iterative single-residue perturbation/relaxation (sPR) until convergence. It incorporates a performance enhancement of only relaxing the ten neighbors of the perturbed residue that have the greatest-magnitude-interaction energies with the perturbed rotamer [33].

The rotamers for every sequence encountered by the genetic algorithm are optimized on each state. This rotamer optimization step, also referred to as “packing,” is the most time consuming step in multistate design. For this reason, we tested several other rotamer packing algortithms.

In addition to the BMEC+sPR algorithm, we examined two simulated annealing algorithms: Rosetta's standard simulated annealing algorithm [34], and another algorithm, the *multi-cool annealing* algorithm. The two algorithms are similar in that they consider single residue rotamer substitutions and use the Metropolis criterion [35] to decide whether to accept or reject each substitution on the basis of the change in energy induced by the substitution.

Briefly, Rosetta's standard annealer starts a geometric cooling trajectory from kT = 100 down to 0.3 in an outer loop. At each temperature, it performs 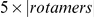 rotamer substitutions. If the energy of the final rotamer assignment at the conclusion of the set of fixed rotamer substitutions for the last three iterations of the outer loop has plateaued, then the temperature is raised back to kT = 100 to begin cooling again. Convergence is determined by comparing the final energy at the conclusion of iteration 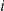 to the average energy at the conclusion of rounds 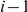, 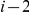, and 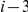; if the energy at round 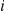 is not less than −1 REU lower than this average, then the energy is considered converged. After 19 iterations of the outer loop, the lowest-energy rotamer assignment encountered thus far is restored, and quenching rotamer substitutions are performed (effectively, kT = 0). The lowest-energy rotamer assignment encountered over the whole trajectory is returned.

The multi-cool annealer differs from the standard annealer in the amount of time it spends at low temperature, especially the amount of time it spends quenching. In an initial geometric cooling trajectory from kT = 10 down to 0.2 performed in 20 iterations, the annealer performs fixed-temperature rotamer substitutions. It performs 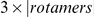 rotamer substitutions at each temperature. Three times in the middle of each set of fixed-temperature rotamer substitutions (sixty times total), the annealer performs *quench-and-restore* operations – saving the current rotamer assignment, performing quenching rotamer substitutions until no new rotamer substitutions can be made, and then restoring the pre-quench rotamers. After cooling to 0.2, the annealer then performs six rounds of cooling from kT = 0.25 to 0.05, using as starting points the 10 lowest-energy quenched rotamer assignments taken from the quench-and-restore steps in the initial cooling from 10 to 0.2. In this second set of cooling trajectories, it performs 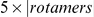 rotamer substitutions at each fixed temperature. Quench-and-restore operations are performed at the end of each set of fixed-temperature rotamer substitutions before the next temperature is assigned. The lowest-energy rotamer assignment encountered over the course of the whole trajectory is returned.

We also examined several hybrid simulated annealing and FASTER algorithms as originally suggested by Allen and Mayo [33]. Most of these algorithms began with a shortened simulated annealing trajectory where, at each temperature, fewer than normal rotamer substitutions were performed, but Rosetta's standard temperature schedule was used. The length of the simulated annealing trajectory relative to the standard trajectory is given by the percentage out front in the name of each algorithm– *e.g.* “5% SimA” represents a trajectory performing 5% as many rotamer substitutions at each fixed temperature. Several of these algorithms are iterated multiple times, and the best energy from all iterations is returned (*e.g.* the 8×(5%SimA+sPR) algorithm iterates through 8 independent trajectories of simulated annealing followed by iterative sPR until convergence). The various rotamer optimization algorithms we tested are compared in Table 1.

**Table 1:** Comparison of Rotamer Optimization Algorithms.

Algorithm	% at	% w/i	% w/i	% w/i	% 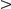	Time	Rel.
	Cons.	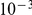 REU	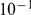 REU	1 REU	100 REU	Avg (s)	Perf.
Standard Annealer	25.3 (26.0)	32.7 (34.5)	42.7 (45.2)	98.5 (91.7)	2E-3 (0)	0.128	1.53×
Multi-cool Annealer	82.8 (85.1)	84.2 (86.6)	87.9 (89.8)	99.2 (97.2)	6E-4 (0)	0.129	1.55×
100% SimA+sPR	81.5 (81.6)	95.1 (96.4)	96.8 (97.3)	99.6 (99.0)	4E-4 (0)	0.165	1.97×
50% SimA+sPR	81.4 (81.6)	94.9 (96.2)	96.5 (97.2)	99.5 (99.0)	5E-4 (0)	0.118	1.41×
20% SimA+sPR	80.9 (81.1)	94.4 (95.8)	96.1 (96.8)	99.3 (98.7)	7E-4 (0)	0.094	1.12×
10% SimA+sPR	80.9 (81.1)	94.4 (95.8)	95.8 (96.5)	99.1 (98.5)	2E-3 (3E-4)	0.091	1.09×
8×(5% SimA+sPR)	84.9 (84.5)	98.8 (99.5)	99.6 (99.6)	99.9 (99.7)	9E-5 (0)	0.481	5.75×
4×(5% SimA+sPR)	84.3 (84.0)	98.2 (99.1)	99.0 (99.2)	99.8 (99.5)	5E-4 (0)	0.255	3.05×
2×(5% SimA+sPR)	82.7 (82.9)	96.4 (97.7)	97.5 (98.1)	99.6 (99.1)	6E-4 (0)	0.143	1.71×
5% SimA+sPR	80.8 (81.1)	94.2 (95.8)	95.7 (96.6)	98.9 (98.4)	2E-3 (0)	0.087	1.05×
8×(2.5% SimA+sPR)	85.0 (84.5)	98.9 (99.6)	99.6 (99.6)	99.9 (99.8)	0 (0)	0.465	5.56×
4×(2.5% SimA+sPR)	84.2 (83.8)	98.0 (98.9)	98.9 (99.0)	99.8 (99.6)	6E-4 (0)	0.248	2.97×
2×(2.5% SimA+sPR)	82.4 (82.5)	96.1 (97.4)	97.2 (97.8)	99.4 (99.1)	1E-3 (0)	0.140	1.67×
2.5% SimA+sPR	80.4 (80.7)	94.0 (95.4)	95.6 (96.3)	98.8 (98.3)	2E-3 (5E-4)	0.085	1.02×
BMEC+sPR	80.0 (80.6)	93.5 (95.2)	95.1 (96.1)	98.5 (97.9)	3E-3 (1E-3)	0.084	1.00×

Fifteen rotamer optimization algorithms were compared by examining the energies they produced and their running times in a head-to-head comparison in optimizing rotamers for 10 K sequences. Details of the optimization algorithms are given in the Methods Section. We defined the “consensus energy” for each sequence examined as the lowest energy found by any of the algorithms. Each algorithm is described by the percentage of the trajectories where it reached the consensus energy, where it reached to within 0.001, to within 0.1, and to within 1 Rosetta energy units (REU) of the consensus energy, (in the columns labeled “% at Cons.”, “% w/i 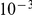 REU”, “% w/i 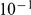 REU”, and “% w/i 1 REU” respectively), the percentage of the trajectories for which it failed to find an energy within 100 REU of the consensus energy (labeled “% 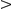
100 REU), its mean running time, in seconds (labeled Time Avg.), and its relative mean running time as compared against the BMEC+sPR algorithm, which was the fastest (labled Rel. Perf.). Parenthetical percentages reflect these frequencies when considering only the subset of sequences with a consensus energy less than −30 REU. 8 K of the 10 K sequences fell into this category.

Over the course of a multistate-design trajectory, rotamer-pair energies are computed as needed and stored in an interaction graph data structure for reuse [36]–[38] instead of all being computed up-front; this saves roughly 25% of the pair energy calculations and the memory needed to store those pair energies. Optionally, the user may set a ceiling on the amount of memory dedicated toward pair energy storage. The interaction graph storing pair energies for reuse honors that ceiling by discarding submatrices of rotamer-pair energies for particular amino-acid pair interactions; it maintains a binary heap of amino-acid-pair-submatrix-access orders and, when discarding a submatrix, chooses the submatrix whose most recent access was furthest in the past. This behavior means that some rotamer-pair energies may be computed multiple times.

#### Fitness Function Definition

The genetic algorithm evaluates the fitness function once for every sequence it examines; the format for the fitness file is geared toward describing how the energies of all the states being modeled should be combined to compute the fitness for a particular sequence, once the rotamers for that sequence have been optimized on each state. This file has two responsibilities: state declaration and fitness-function specification. The fitness-function-definition file format provides seven commands to meet these two responsibilities. They are referred to as “commands” as our software effectively defines a programming language for multistate design. It is possible to specify expressions using basic arithmetic such as addition, subtraction, and multiplication as well as with min and max functions for identifying the minimum or maximum values from a vector of values. The min function is particularly useful for finding the lowest scoring state from an ensemble of negative states. The seven commands are described in detail in the Supporting Information File S1 and examples of how they are used in the two design tasks for this paper are given as well.

#### Redocking

**Figure 1:**
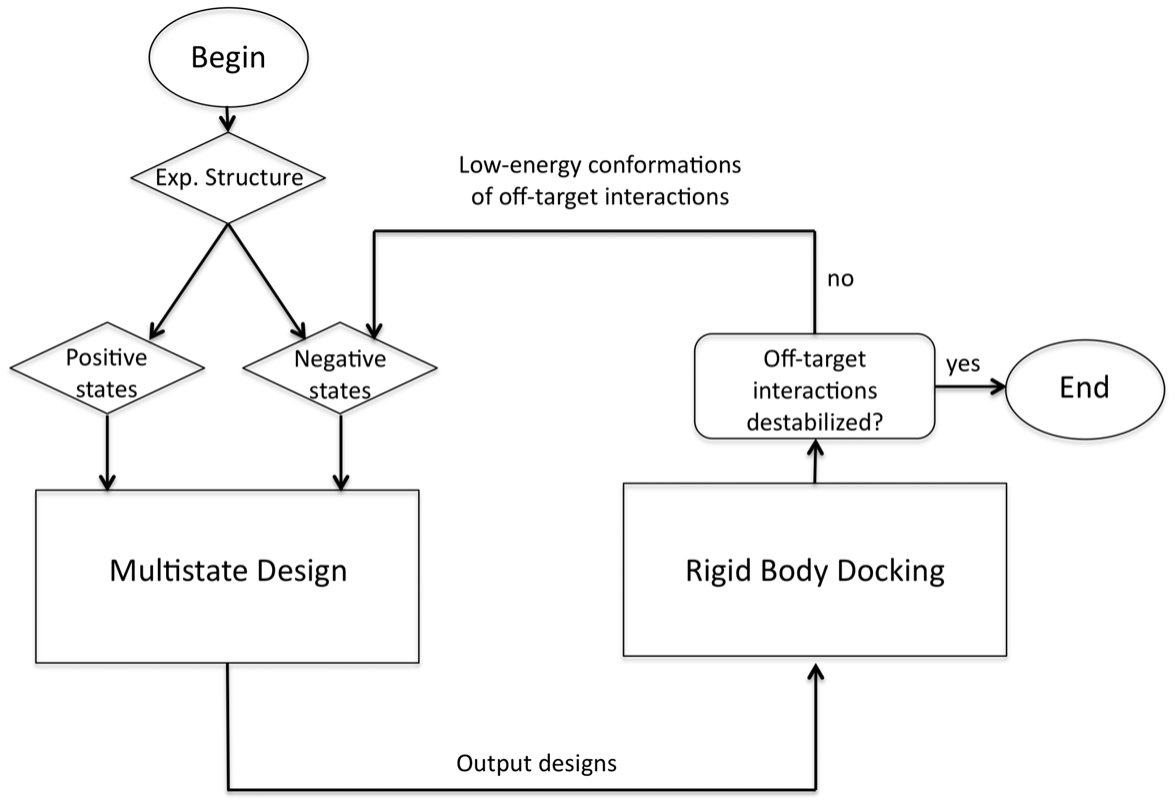
Iterative multistate design. This flow chart summarizes the way we used rigid-body docking to expand the set of conformations that we designed against. A “round” of multistate design is a single execution of the multistate design executable with a given set of input positive and negative states. The first round begins using the experimentally determined structure(s) (either from x-ray crystallography or NMR) for both positive and negative states; subsequent rounds include low-energy conformations for the undesired interactions in the set of negative states generated by redocking the models for those interactions generated by prior rounds of multistate design.

After each round of multistate design, we redocked the negative states to find alternate low-energy conformations, and then designed against these alternate docked conformations in subsequent rounds (Figure 1). We used the *dock_pert* rigid-body docking protocol [39] that begins with a small random rigid body perturbation of an initial docked conformation. Starting from the output structures from multistate design, we split the two chains, packed each chain separately, and concatenated the packed structures. This step relieved intra-chain collisions frequently present in the negative states which the shorter *docking_local_refine* protocol seemed willing to leave intact. These structures were then fed as input for fifty trajectories of the *dock_pert* protocol. The lowest energy docked conformation of these fifty was split, its chains packed individually, and the 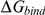 was calculated as the difference in energy of the bound and unbound chains. All parameters and scripts for these redocking and packing protocols are given in the Supporting Information File S1.

### Detailed Workflow

Both the heterodimerization task and the orthogonal interface redesign task were similar in their overall setup. In both cases, one protein interface was desired and two protein interfaces were undesired. In both cases, an overarching iterative process was used where, starting with crystal and NMR structures as models for our positive and negative states, we ran multistate design to generate candidate sequences and then ran rigid-body docking to relax the structures for the negative states (Figure 1). Sequences were evaluated on the basis of the binding energy of both the desired and undesired interactions after redocking.

#### Heterodimerization Task Workflow

*Choosing what to design:* The dimeric hepatocyte nuclear factor 1-alpha from T. Thermophilus (PDB ID: 1USM) was selected as a worthy heterodimerization target. The hydrophobic residues at the center of the dimerization interface, F21, A24, L25, A28, G32, L44, V46 and W48, were selected as candidates for redesign. The (mostly polar) residues at the boundary of the interface, R22, N29, D36, H41, P42, R43, T45 and E47, were prevented from being designed but were allowed to pack. In general, it seems that Rosetta does not do well at the design of polar residues at protein interfaces [16], so we restricted our mutations to the hydrophobic core of the interface. This selection allowed 8 mutations per monomer for 16 designable positions total.

*Input file preparation:* In this task, it is convenient to talk about two chemical species, the A monomer and the B monomer, which can form five possible arrangements: the A monomer alone, the B monomer alone, the AB heterodimer, the AA homodimer, and the BB homodimer. States representing all five arrangements were defined with PDBs, correspondence files, and secondary resfiles for each. The 16 positions in the sequence optimized by the genetic algorithm were arranged with the odd elements assigned to the “A” chemical species, and the even elements assigned to the “B” chemical species. The sequence “HfaaGMMagRlVMLFF,” for example, would describe a heterodimer where the A monomer has the mutations F21H, (A24), L25G, A28M, (G32), (L44), V46M, V48F, and the B monomer has the mutations (F21) (A24) L25M (A28) G32R L44V V46L V48F – where positions with lower case letters in the first sequence, or in parenthesis in the second expansion, represent the wild-type sequence. This convention for residue correspondence is arbitrary and, for the sake of the search through sequence space itself, irrelevant. The correspondence file for the AA states listed both chain 1 and chain 2 residues as corresponding to the odd positions, the correspondence file for the BB states listed both chain 1 and chain 2 residues as corresponding to the even positions, and the correspondence file for the AB states listed chain 1 residues as corresponding to the odd positions and chain 2 residues as corresponding to the even positions. The same secondary resfile was used for the AB, AA, and BB species.

*Fitness function definition:* The fitness function for this design task examined the difference in binding energies between the the AB heterodimer and the AA and BB homodimers. If the variables 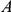, 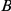, 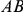, 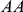, and 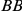 hold the best energies over all states in the A, B, AB, AA and BB arrangements given a particular sequence which has been threaded on to all states, then the fitness function we optimized was
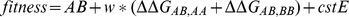


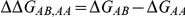


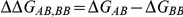


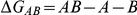


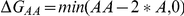


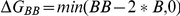
where, 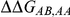 and 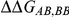 represent the *binding-energy gaps*, and the *binding-energy-gap weight*, 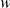, balances the total energy of the heterodimer (AB) and the binding energy gaps. This weight was varied between 1 and 6, to generate a spectrum of design results. The sequence constraint energy, 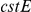, is described in the next paragraph. Note that we cap the maximum binding energy for the negative states at 0. We describe this cap in greater detail in the Discussion section.

The fitness function included a sequence-constraint term, 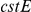 in the fitness expression above, that contained two features: a homodimer penalty and a minimal mutation bonus. The homodimer penalty gave a positive score representing the number of positions on A and B that were assigned the same amino acid, clipped at 0 if the number of identical positions were 6 or fewer, and stepped by 5 REU for every successive identical amino acid pair. The mutation penalty added a penalty of 1 REU for each mutation beyond the first five to either chain. The homodimer penalty was intended to push the search in sequence space away from homodimers, which we found in preliminary testing would sometimes get designed; the mutation penalty was added to bias the search to the minimal set of mutations required to accomplish the task. The full sequence constraint definition file for the heterodimerization task is included in the Supplemental Information.

As a control, we simulated single-state design with our multistate design software by setting the weight on the binding-energy-gaps to zero. The sequence-constraint term remained present in the fitness function to favor the design of heterodimers over homodimers. The remainder of the protocol following multistate design (packing and redocking) remained unchanged.

*Iterative design and docking*. We performed four rounds of design and docking. In each round, we ran one multistate design trajectory for each combination of binding-energy-gap weights (of which there were six) and conformation for the heterodimeric complex (of which there were seven), where, in addition to the crystal structure of the homodimer, the other six conformations were generated by performing rigid body docking on the wildtype homodimer and selecting conformations with sub-angstrom RMS from the wildtype structure. Thus, forty two multistate design trajectories were performed in each round. Following each multistate design trajectory, we ran 50 docking trajectories for both the positive and negative states using the dock-pert protocol. After redocking, the conformations of the homodimers (the negative states) which had binding energies 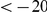 REU were identified. These structures were filtered to select a subset with mutual C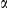 RMS (without superposition) 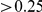 Å, and the resulting set of structures was then used for the negative states in the next round.

*Job management:* Each batch of jobs was composed of two main features: the set of PDB files defining the states which should be optimized (the state version), and the set of residues which were allowed to redesign and pack on each of the states (the design definition). Each batch ran separate jobs for each combination of models of the positive state (the heterodimer) and weight, 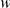, on the binding-energy-gap bonus. A Python2.6 script created the set of files necessary for a single batch of multistate design jobs. This script, the set of input files necessary for it, and the command lines we used to execute this script are provided in the Supporting Information File S1. Following each round of multistate design, we redocked the homo- and heterodimers using the RosettaScripts executable [40], and then packed the monomers using the fixbb (fixed-backbone design) executable. All simulations were performed with SVN revision 39931 of the Rosetta3 source code and SVN revision 39914 of the Rosetta3 database.

#### Orthogonal Interface Redesign Task Workflow

*Choosing what to design:* For the orthogonal interface redesign task, we chose to redesign the interactions in the Ral signaling network. Ral is a small GTPase protein that is involved in a wide variety of cellular functions including endocytosis, transport and tethering of secretory vesicles to the plasma membrane, regulation of transcription, and maintenance of the cytoskeleton, among many others [41]. Ral has also been shown to be important for Ras-mediated tumorigenesis and tumor cell metastasis [42], [43]. Ral exists in two isoforms, RalA and RalB, which are 82% identical, and has five known effectors: RalBP1, Sec5, Exo84, Filamin, and ZONAB. The Ral signaling network is an attractive model system for testing the multistate design protocol for two reasons. First, structures of RalB in complex with RalBP1 (PDB: 2KWI) [44] and RalA in complex with Sec5 (PDB: 1UAD) [45] and Exo84 (PDB: 1ZC3) [46] have been solved. Second, some amino acid positions on Ral are contacted by more than one effector, making orthoganol interface redesign nontrivial. If there were no overlap between the various interfaces, then simply converting all the off-target interface surfaces to arginine would likely have produced the desired set of interactions. We should point out that, although 2KWI is actually the structure of the interaction between RalB/RalBP1, RalA and RalB have complete sequence identity at all of the interface positions considered in this study. Henceforth, we refer to this complex as Ral/RalBP1 to imply that the RalB structure is used to model the RalA structure in complex with RalBP1.

We decided to redesign RalA to retain its affinity for RalBP1, but to remove its affinity for Sec5 and Exo84. Two different setups of the redesign task were performed with multistate design. In the first setup, we selected residues on RalA that we thought could destabilize the interface between RalA/Sec5 and RalA/Exo84 without disturbing the Ral/RalBP1 interaction. Residues L14, Y36, Q63, and E73 were chosen because they interact with either Sec5 or Exo84 but not RalBP1. Additional positions that were designed included E38, K47, A48, D49, S50 and R52. Mutagenesis studies have indicated that these residues affect Exo84 and Sec5 binding [46]. Note that RalA residues A48, D49, S50 and R52 are in close proximity to RalBP1; mutations to these residues would impact both RalA's interactions with Sec5 and Exo84 and its interactions with RalBP1. Design at these positions is therefore non-trivial.

For the second setup of this task, we excluded some of the already-characterized specificity-determining positions and also allowed more residues at the Ral/RalBP1 interface to be designed. From the structures of the Ral-effector complexes, mutations on Ral that disrupt binding to each individual effector have already been identified. For example, the D49N mutant of RalA disrupts binding to RalBP1 but not Sec5 or Exo84, and the D49E mutant disrupts binding to Sec5 and Exo84 but not to RalBP1 [47], [48]. Similarly, the mutations E38R and A48W have been shown to destroy binding with Sec5 and Exo84, respectively [46]. In order to make the design task more challenging, we left these positions fixed to their native amino acids. Additionally, for this setup we allowed more residues at the interface between Ral/RalBP1 to be designed, to see if the binding energy of the positive state could be further improved. Together, these changes expanded the number of designable residues from 8 to 16. The residues allowed to change in this setup were L14, K16, Y36, K47, S50, R52, Q63, D65, L67, E73, D74, Y75, A77, I78, N81, and Y82. With this design definition, we hoped to identify new specificity-conferring mutations for Ral.

*Input file preparation:* An important step in setting up the orthogonal interface design is obtaining reliable starting structures for design. The crystal structures for the RalA/Sec5 and RalA/Exo84 interactions were packed and minimized using Rosetta to obtain low energy models. The structure of Ral/RalBP1 is more difficult to handle in this way because its PDB entry is an ensemble of NMR models which vary in conformation considerably. All of the models in the 2KWI structure were separated into individual models, packed, and minimized. We then chose the four lowest-energy structures, models 1, 15, 29 and 30, and redocked them with Rosetta [39]. Model 30 produced the best docking funnel and binding energy, and did not substantially change the conformation of the interface (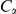 RMSD
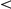
2.0 Å). The lowest-energy docked conformation starting from model-30 was used for the Ral/RalBP1 complex.

For convenience it is useful to describe the proteins modeled as four different chemical species. Each protein monomer is described as A (RalA), B (RalBP1), C (Sec5) or D (Exo84). The three dimers can be described as as AB (Ral/RalBP1), AC (RalA/Sec5) or AD (RalA/Exo84). The AB species refers to the best packed, minimized, and docked model 30 from 2KWI while AC and AD refer to the packed and minimized 1UAD and 1ZC3 structures respectively.

*Fitness function definition:* The fitness function used for orthogonal interface design was constructed to use the binding energy of the desired Ral/RalBP1 interaction and the binding energies of the undesired RalA/Sec5 and RalA/Exo84 interactions. Using the nomenclature described above, with 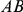, 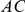, 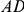, 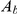, 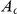, 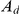, 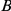, and 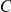, representing the energy of each of the corresponding dimer or monomer under a particular sequence assignment (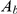 representing the energy of the Ral backbone taken from the Ral/RalBP1 structure, 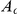 representing the energy of the RalA backbone taken from the RalA/Sec5 structure, and 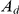 representing the energy of the RalA backbone taken from the RalA/Exo84 structure), the fitness function we minimized was
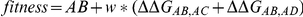


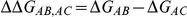


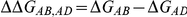


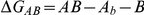


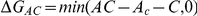


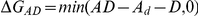
where 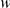 is the weight used to balance the total energy of the AB complex and the binding-energy-gaps for AC and AD. The weight was varied in independent runs between 1 and 12 in increments of 1. We capped the binding energies of the negative states at 0 as we did in the heterodimerization task. We computed binding energies by comparing the energies of the dimers with energies of the monomers sharing the same backbone conformations; this meant modeling extra states (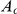 and 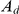), but gave more reliable results than if we had only modeled the 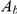 monomer. The Discussion section raises this point again.

We ran two separate single state design (SSD) tasks as controls for this method. This protocol optimized the binding energy of AB while ignoring the binding energies of AC and AD. In the first control run, we allowed design of all of the residues included in the multistate design setup-scheme 2 (SSD 1). In the second control run (SSD 2), we designed only residues on RalA that are at the interface with RalBP1 in an effort to mirror redesign of only one complex. The set of residues in this case were as follows: K16, A48, D49, S50, R52, D65, L67, N81, Y82, R84, S85, G86. We used the same protocol as above except the weight (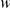) of the fitness function is set to 0 to force the design algorithm to ignore binding of AC and AD.

*Iterative design and docking*. Iterative rounds of design and docking were performed for each setup of the othogonal interface redesign task. Two rounds of design and docking were performed for setup one, and three rounds were done for setup two. Each round for setup one consisted of twelve multistate design trajectories, one for each value of the binding-energy-gap-weight. For setup two, the number of design trajectories in each round varied. The number of design trajectories in rounds one, two and three were twelve, eighteen and thirty-six, respectively. The additional trajectories were obtained by 1) varying the binding-energy-gap-weight between 1 and 12 in a smaller increment of 0.5 and 2) running multiple trajectories for the same binding-energy-weight. The number of trajectories per round for setup two was increased to better see how the binding energy error changed between rounds.

*Job management:* A similar python script as described for the heterodimerization task was used to prepare batches of multistate design jobs and can be found in the Supporting Information File S1. All simulations were performed with SVN revision 39931 of the Rosetta3 source code and SVN revision 39914 of the Rosetta3 database. Sequence logos were created using WebLogo v.2.8.2 [49].

## Results

### Comparison of Rotamer Optimization Algorithms

The outer loop of our multistate design algorithm explores sequence space, and for each sequence it examines, it performs a rotamer optimization of that sequence for each of the states. As the vast majority of the running time is spent in rotamer optimization, we compared a set of optimization algorithms to examine reliability and speed. We tested fifteen subtle variations and combinations of the FASTER and simulated annealing algorithms (see the Methods Section for details) in a head to head comparison where we sampled 10 K sequences encountered in the course of a multistate design trajectory. The fitness function we used in this trajectory optimized the total energy of a single state for the 32 interface residues from the 1USM heterodimerization task. Running times excluded the expense of computing rotamer-pair energies, but did include the expense of initializing a small data structure to store those energies – this extra expense was well worth the time as it improved cache efficiency greatly. Running times were measured on an Intel i7 920, 2.67 GHz processor with 12 GB RAM using a single thread.

Table 1 summarizes the comparison between these fifteen algorithms. For each sequence in the 10 K we repacked, we compared the energy computed by each of the packing algorithms to the lowest-energy produced by any of the algorithms, which we defined as the consensus energy. It should be noted that the consensus energy is not the same as the energy from the global-minimum energy conformation (GMEC) [50]. None the less, comparing energies against the consensus energy is a reasonable way to show that packing algorithms are imperfect. Table 1 describes the quality of each packing algorithm by its frequency of reaching the consensus energy, and its frequency of reaching to within 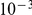, 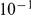, and 1 REU of the consensus energy. It also describes these frequencies when considering only the subset of sequences with a consensus energy less than −30 REU. (The best energies were found in the neighborhood of −90 REU.) This subset of sequences is meant to reflect those which are relatively collision free and whose energies the user might be particularly interested in measuring accurately. In particular, if a negative state has an available collision-free rotamer placement, and the packing protocol fails to find this placement, then the fitness function will appear better than it should. The Discussion section addresses this point again.

The BMEC+sPR algorithm, which is the one we decided to use in our design simulations for this study, proved to be the fastest, and was further than a tenth of an energy unit from the consensus energy only 3.9% of the time when considering the collision-free placements. In the vast majority of the cases, all the FASTER algorithms converged to the same energy or, when they arrived at higher-energy assignments, were within a tenth of an energy unit of the consensus energy. None of the algorithms arrived at the consensus energy 100% of the time; this suggests that there will always be some noise in the fitness for any given sequence. For states whose energies we were particularly interested in getting correct (in particular, the positive states and the monomer states), we declared multiple copies of those states in their state files, packed them in duplicate (and on separate processors), and then took the best energy. This is somewhat similar to using the 2×(5%SimA+sBR) algorithm, except that the extra effort of packing twice can be focused on a small subset of all states, providing higher accuracy for those states without increasing the overall running time of the trajectory.

Surprisingly, Rosetta's standard simulated annealing algorithm was further than a tenth of an energy unit from the consensus energy in over half the trajectories. The multi-cool annealer, on the other hand, was within 0.1 REU of the consensus energy nearly 90% of the time, and at the consensus energy as often as any of the FASTER based algorithms. This result is worth noting for single-state design applications. A key difference between FASTER and simulated annealing is that FASTER examines every rotamer-pair energy while simulated annealing examines a small minority. In single-state design, the expense of computing all of the rotamer-pair energies is considerably greater than the expense of optimizing rotamers once the energies are computed, so a technique that performs as well as FASTER while computing many fewer rotamer-pair energies would, on the whole, be preferable [38].

### Heterodimerization task

**Figure 2:**
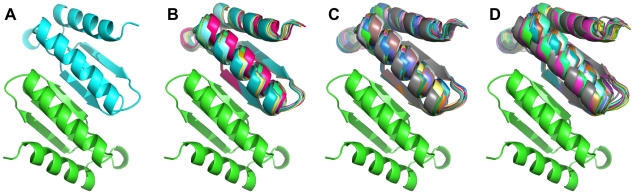
Iterative expansion of the negative state set. A) the original 1USM homodimeric complex used as both the positive and negative states in the first round of multistate design, B) the thirteen negative states used in the second round, C) the forty one negative states used in the third round, and D) the sixty seven negative states used in the fourth round.

Out iterative protocol for designing a heterodimer began by using the crystal structure of the homodimer to model the negative-state conformations and the heterodimer (Figure 1). After the first round of design, the homodimeric forms were redocked. The redocked homodimers with the lowest binding energies were used as alternate conformations for the second round of design. This process of design, redocking and feeding in the low-energy docked conformation back into the next round of design continued for a third and a fourth round (Figure 2). The fitness function used in multistate design and the residues allowed to redesign are described in the Methods section.

**Figure 3:**
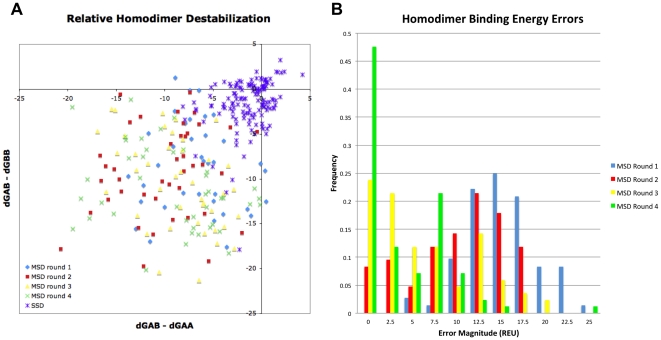
Binding energies differences for the heterodimerization redesign task. A) The distribution of 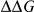
s for the homodimers *vs* the heterodimers comparing single state design (SSD) against multistate design (MSD). Binding energies were computed by redocking each of the complexes, and computing the difference between the lowest-energy from docking and the energies of the unbound monomers after their interface residues were allowed to pack. B) Histogram of the homodimer binding energy errors for each of the four rounds of multistate design. Errors were measured as the difference in the binding energies as computed by multistate design and as computed after redocking.

We compared this iterative design strategy against the simpler single-state design strategy, using the multistate design algorithm, but optimizing only for the energy of the heterodimeric state (Figure 3A). As Havranek and Harbury observed [26], single state design fails to simultaneously destabilize both homodimeric species, though, by luck, it may destabilize one of the two. In contrast, multistate design is able to produce the desired destabilization of the homodimers relative to the heterodimers. Such designs are represented in the lower-left cornert of Figure 3A. Table 2 highlights a few designs where both homodimers were destabilized by at least 10 REU relative to the heterodimer and the heterdimer's total energy had not been overly compromised relative to the native (total energy
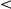
−298 REU).

**Table 2:** Selected heterodimeric sequences.

Design No.	Designed sequence	Design Round	Total Energy	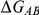	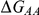	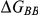
wt	falaglvw/falaglvw	-	−307.3	−23.4	-	-
1	HalaRVAF/HaGMglMF	1	−298.5	−25.6	−15.6	−12.4
2	HaGMgAvF/faRaRlIF	1	−304.1	−26.4	−13.4	−10.8
3	fSlaRMvY/HaGMglAH	2	−298.5	−24.6	−12.4	−4.8
4	HaGMgTvF/YaMaRMIw	2	−299.7.9	−24.7	−12.0	−9.3
5	faRaRlIw/HaGMgAvF	4	−305.0	−26.5	−7.9	−14.4
6	faRaRVvY/HaGMglMF	4	−302.8	−27.5	−8.9	−11.9
7	HaTGglMF/falaHlvw	*	−304.4	−26.9	−17.4	−16.6
8	SaQaglvY/falaQlvF	*	−306.5	−23.2	−17.9	−15.2
9	HaGMglEF/falaRlvw	*	−302.3	−26.0	−11.9	−13.3

Chain A and chain B sequences for selected heterodimer designs, their total energies, and binding energies, in REUs. The first six designs were selected based on the total energy of the heterodimer (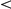
−298 REU) and that both homodimer binding energies were destabilized relative to the heterodimer binding energy by 10 REU, the last three designs originated from the “fitness2” fitness function, and though they generally had smaller binding energy gaps between the heterodimer and the homodimers, their heterodimer total energies were generally better than designs produced by the first fitness function. The models for these designs output by multistate design and those output by docking are included in the Supporting Information File S2.

Our results also suggest that iterative negative design improves multistate design. We defined the error in multistate design as the difference between the homodimer binding energies computed by multistate design and the homodimer binding energies computed after redocking, and measured this error for each of the four rounds of design. The distribution of binding-energy errors shifted to smaller values as more conformations of the negative states were included. The mean binding energy errors for the four rounds were 16.5, 11.5, 7.4 and 4.8 REU. This shift is evident in the histogram of binding energy errors, as shown in Figure 3B. After four rounds, there were sixty seven conformations used to model each of the two homodimer interactions. These simulations, counting the positive states, included 140 states total.

The search through sequence space which the genetic algorithm performs is NP-Complete for the same reasons that the search through rotamer space is NP-complete [51]. Though the genetic algorithm seems to perform well, we do not expect it to find the absolute best sequence for a given fitness function. For this reason we ran the entirety of the multistate design algorithm several times. We are thus not surprised that some of our trajectories generated in later rounds produced worse results than some of the best results from the earlier rounds. Similarly, the inaccuracy in the early rounds of design did not preclude multistate design from serendipitously finding some sequences that both destabilized the homodimers and generated low-energy heterodimers (as several of the best designs in Table 2 came from early rounds). That said, this experiment does suggest that accuracy in negative design does increase with the expansion of conformational sampling for the negative states. Indeed the problem with designs that were generated in later rounds tended to be that the total energies of the heterodimers were low, even though their binding energies were quite good. This was a consequence of the set of binding-energy-gap weights we examined in this study. The values we scanned for the binding-energy-gap weight, 1 through 6, favored the binding energy of the heterodimer over its total energy. That is, the fitness function can be rewritten as 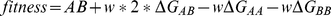. Therefore, any weight, 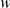, above 0.5 favors trading 1 REU of heterodimer total energy for 1 REU of heterodimer binding energy, and all the weights we scanned were above this break-even point of 0.5. We also ran a set of trajectories using weights between 0.1 and 0.5; however, these trajectories failed to produce designs that sufficiently destabilized both homodimers.

In response to this weakness in our original fitness function, we ran a series of mulitistate design trajectories using an alternate fitness function:
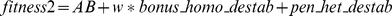


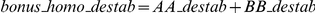


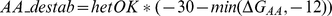


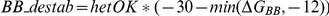


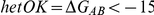


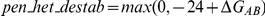
where the terms 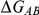, 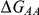, and 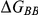 have the same definition as in the original fitness function. Fitness function 2 aimed to stabilize the heterodimer, to preserve the binding energy of the heterodimer near the wildtype value of −24 REU, and to destabilize the homodimer binding energies toward −12 REU. The weight, 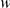, applied to the bonus for destabilizing the homodimers was sampled at values 0.5, 0.75, 1.0, 1.5, and 2.0. The purpose of the 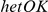 variable was to avoid applying a homodimer-destabilization bonus to sequences where the heterodimer did not have a good energy; this decision was made to avoid creating local minima in the fitness-function landscape in regions that were distant from the kinds of sequences we were seeking. The main feature of this second fitness function is that, because it did not reward the heterodimer binding energy beyond −24 REU, it did not seek to trade total energy for binding energy and thus produced designs with better heterodimer total energies than the first fitness function. On average, the designs produced with this fitness function had a total energy of −300.7 REU, whereas the designs from round four produced with the original fitness function had an average total energy of −288.9 REU. One of the sequences produced with the original fitness function suffered from a pitfall where a packing failure in one of the monomers produced an apparent binding energy of −70 REU for the heterodimer and binding energy gaps of −30 and −50 REU; the fitness function rewarded this sequence heavily, in spite of the fact that the total energy of the heterodimer had been destabilized to −158 REU. Excluding this failed design improves the average heterodimer energy for round-four designs to −293.3 REU, which is still several energy units worse than those produced with the second fitness function. The second fitness function both avoids the pitfall of rewarding the destabilization of the (negative state) monomers, and of overly preferring heterodimer binding energy to heterodimer total energy.

### Orthogonal Interface Redesign

As another test of the multistate design protocol, we decided to redesign specificity in the Ral signaling network. Our design goal for this task was to redesign RalA so that it would only interact with RalBP1 and not with Sec5 or Exo84. For the first setup scheme, any position on RalA that we thought could be used to improve specificity for RalBP1 was allowed to change. This set included positions which have already been shown to be important for specificity with the various Ral effectors. The results from setup-scheme 1 are shown in Table 3. The predicted binidng energies given in this table reflect energies computed after redocking all of the complexes output by the design protocol. After only one round of design and docking, many designs showed large destabilizations to the RalA/Sec5 and RalA/Exo84 interfaces while maintaining native-like Ral/RalBP1 binding energies.

**Table 3:** Selected orthogonal interface sequences from setup-scheme 1.

Design round	Design no.	Designed sequence	Total Energy	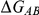	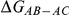	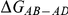	RMSD to native		
							AB	AC	AD
–	wt	lyek adsr qe	−463.0	−22.6	2.7	8.7	–	–	–
1	1	DWQE Rdsr ED	−463.6	−24.4	−16.7	−18.5	0.1	1.4	1.7
1	2	DWQE Rdsr ED	−463.7	−23.7	−16.2	−18.3	0.1	4.7	5
2	3	DKWW YdsI Te	−460.2	−21.6	−15.7	−17.7	0.2	2.5	5.2
1	4	DRQE RdsM HD	−461.3	−23.8	−17.9	−15.4	0.1	2.3	2.2
1	5	DRQE RdsM HD	−463.4	−23.0	−12.8	−20.4	0.1	1.1	4
1	6	DRQE RdsM HD	−463.6	−23.7	−14.5	−18.6	0.1	2.6	2.4
2	7	DKWW YdsI TD	−461.0	−22.3	−14.3	−18.5	0.1	3.7	5.8
2	8	ELQW FdsF Ee	−457.6	−22.1	−16.3	−15.7	0.1	2.4	4.3
1	9	DWQE Rdsr ED	−461.1	−23.5	−15.0	−16.6	0.1	1	3.3
2	10	DKWW RdsI SW	−458.8	−22.0	−14.5	−17.1	0.2	5.2	5.3

Sequences, energies (in REUs), and RMSD's of designs created multistate design (MSD). All of the MSD designs shown have binding energy gaps between the positive and negative states greater than 10 REU.

It was reassuring to us to see that the multistate protocol recapitulated some known specificity-changing mutations. Lysine-47 in wild-type RalA was mutated most often to glutamic acid. Fukai *et al.* found that the K47E mutation weakens binding to Sec5 10-fold and to Exo84 about 40-fold [45]. Alanine-48 of RalA, part of the switch I region and at the interface of all three effectors, is mutated to arginine in all of the round one and most of the round two designs. A tryptophan mutation at this residue was previously found to decrease binding of Exo84 but had no effect on Sec5 [45]. We suspect that this tryptophan's effect on Exo84 binding is due to steric repulsion and hypothesize that the designed arginine at this residue would work equally well. Replacing arginine-52 with a tryptophan decreases Sec5 binding 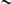
100-fold while having no effect on Exo84 binding [45]. Rosetta did not design any tryptophanes at this position, but did select other bulky hydrophobic residues including phenylalanine and methionine. Not all specificity changing mutations were recovered. The multistate design protocol failed to identify the destabilization of both Sec5 and Exo84 binding induced by the glutamic acid mutation at residue 49 [48]; instead, it chose the wild-type aspartic acid at this position in all of the designs.

**Figure 4:**
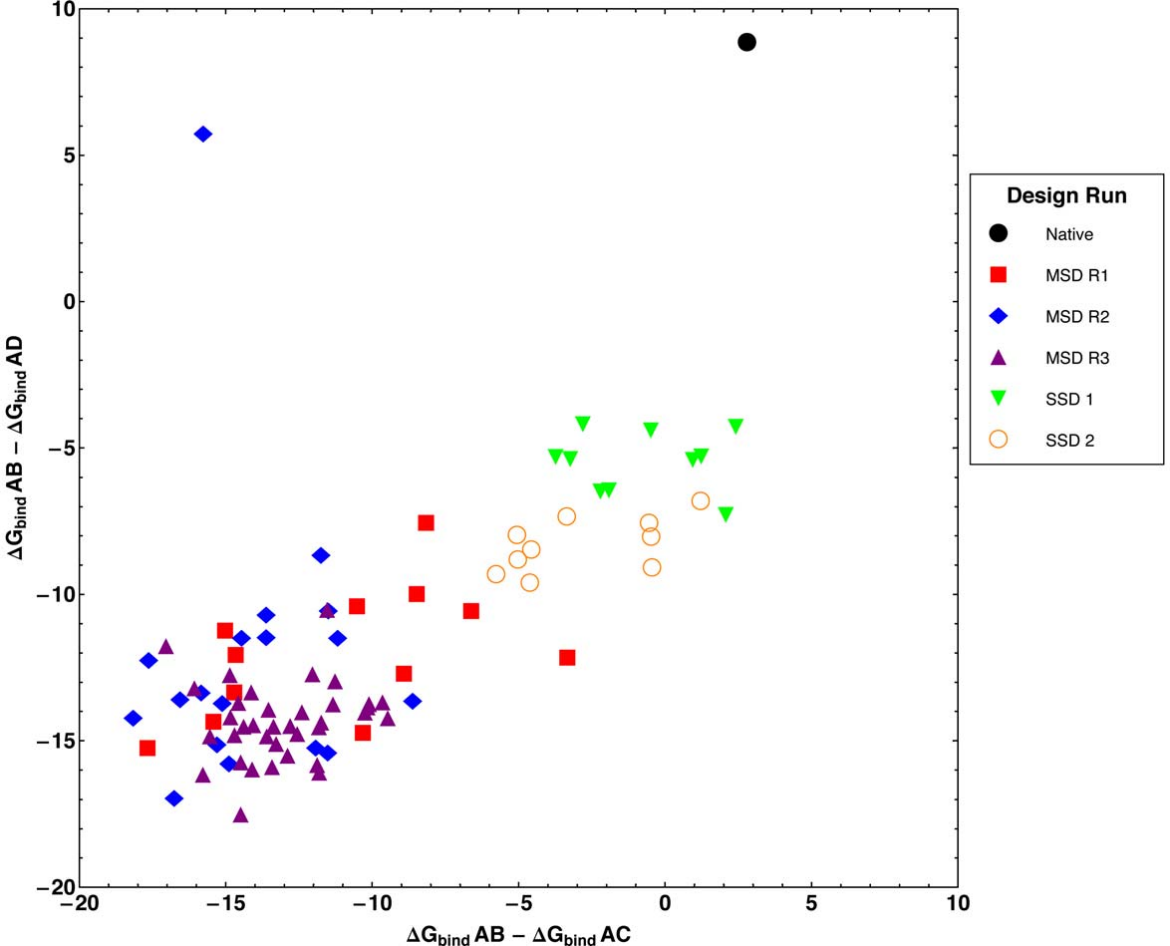
Binding energy differences for the orthogonal interface redesign task. Binding energy differences between the positive state AB (Ral/RalBP1) and negative states AC (RalA/Sec5) and AD(RalA/Exo84) following multistate design (MSD) and single state design (SSD). Binding energy differences between the native AB and AC, and AB and AD states (black) are shown for reference. Consecutive rounds of MSD (red, blue, and purple) on protein A residues, listed in Methods, decrease the binding energy to C and D by a larger magnitude than SSD. Two different methods of SSD are shown: SSD 1 (green) allows design on the same residues as MSD, and SSD 2 (orange) allows design on residues that are at the AB interface. Neither of the SSD methods explicitly disfavor binding to C or D. AB binding energy maintained, in all cases, between −22.0 and −25.0 REU.

In the second setup scheme, multistate design found many new RalA mutations that have not been previously characterized. As described in the Methods section, the difference between the two setup schemes was the set of residues allowed to change. Briefly, the second scheme included all of the positions varied in the first scheme except positions 38, 48, and 49, and the second scheme allowed more residues at the Ral/RalBP1 interface to vary. The results from this setup scheme and the results from the single-state design control runs are shown in Figure 4. Again, multistate design succeeded at destabilizing the undesired interactions better than could be achieved simply by positive design for a single state, as is shown by the points in the lower left quadrant of Figure 4. Single state design produced designs that have good binding energies for the target interface Ral/RalBP1, but they also have good binding energies for the RalA/Exo84 interaction (Table 4). Only the designs created with multistate design showed significant destabilization of both off-target interactions, RalA/Sec5 and RalA/Exo84. As in the heterodimerization task, we calculated the difference in binding energy gaps between what was reported by multistate design and what was reported by docking. The mean binding energy errors for rounds one, two, and three were 36.7, 11.4, and 7.2 REU showing that multistate design's accuracy at negative design increases as the number of negative states increases.

**Table 4:** Selected orthogonal interface sequences from setup-scheme 2.

Design no.	Designed sequence	Total Energy	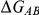	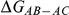	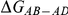	RMSD to native		
						AB	AC	AD
wt	lkyk srqd ledy ainy	−466.2	−22.5	2.8	8.8	–	–	–
ssd,1	VRyE srEd YDKy SRDy	−472.5	−24.0	−2.2	−6.5	0.1	0.3	0.2
2	VRyF srEd FDEH STDy	−469.1	−23.7	−1.9	−6.5	0.1	0.2	0.3
3	VRyE srEd YDKy SRDy	−472.2	−23.3	−0.5	−4.4	0.1	0.1	0.2
4	VRyE srEd YDKy SRDy	−472.1	−23.3	−3.7	−5.3	0.1	0.2	0.2
5	VRyE srEd YDKy SRDy	−472.0	−23.1	−3.2	−5.4	0.1	0.1	0.4
6	VRyE srEd YDKy SRDy	−471.9	−23.0	−2.8	−4.2	0.1	0.1	0.1
7	VRyE srEd YDKy SRDy	−470.8	−22.9	0.9	−5.4	0.1	0.1	0.1
8	RRyE sHEE YDKy SRDy	−471.1	−22.6	2.1	−7.3	0.2	0.1	0.9
9	VRyE srEd YDKy SRDy	−471.1	−22.4	1.2	−5.3	0.1	0.1	0.2
10	VRyE srEd YDKy SRDy	−471.1	−22.3	2.4	−4.3	0.1	0.1	0.1
msd,1	WFKF sFSG lKQH SWDy	−460.4	−25.9	−17.0	−11.8	0.1	4.5	0.8
2	EHKN sFEd YGRE STDF	−465.5	−25.0	−15.8	−16.2	0.1	6.6	2.2
3	RRTQ sLVV YKRE SSDF	−465.6	−25.0	−12.9	−15.5	0.0	6.0	1.3
4	DHTF sITd lKNQ SWDy	−463.2	−24.7	−10.1	−13.8	0.1	0.6	1.0
5	EHKT sFES lKSR SLDy	−462.6	−24.5	−14.6	−13.7	0.1	6.2	0.4
6	EHKT sFES lDSR SLDy	−464.5	−24.5	−15.5	−14.9	0.1	6.2	0.4
7	KRRF sLVV lKQH SWDy	−462.5	−24.2	−14.4	−14.5	0.1	0.9	3.3
8	EHKT sFES lDSR SLDy	−464.3	−24.1	−14.8	−14.2	0.1	5.4	0.6
9	EHKT sFES lNSR SLDy	−464.0	−24.0	−14.5	−15.8	0.1	5.4	1.8
10	EHKG sFEd lKQH SRDy	−464.1	−23.8	−14.9	−12.8	0.1	6.3	4.8

Sequences, energies (in REUs), and RMSD's of designs created with single state design (SSD) and multistate design (MSD). All of the MSD designs shown have binding energy gaps between the positive and negative states greater than 10. None of the SSD designs are predicted to have this amount of specificity.

**Figure 5:**
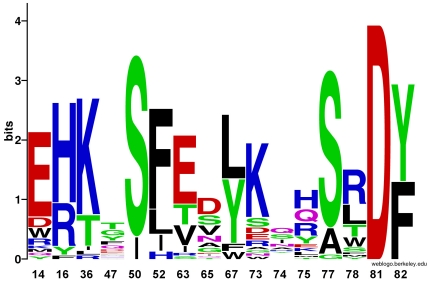
Sequence propensity of RalA residues from multistate-design. Sequence logo of the designs produced by multistate design in setup-scheme 2 for the RalA orthogonal interface redesign task. Positions 50, 67, and 16 showed preferences for amino acids that stabilized the RalA monomer or that stabilized the Ral/RalBP1 complex. Positions 36 and 52 showed preferences for amino acids that destabilized the RalA/Sec5 interaction; positions 14, 77, 78, and 81 showed preferences for amino acids that destabilized the RalA/Exo84 interface. Positions 47, 73, 74 and 75 displayed no clear preferences, except for non-wildtype amino acids, as the native amino acids formed favorable contacts with either Sec5 or Exo84.

The designed amino acids from this second design setup fell into three categories: those which appeared important for RalA stability or RalBP1 binding (often including the native amino acid), those which appeared to destabilize either Sec5 binding or Exo84 binding, and those which showed no clear preference. The sequence profile of these designs is given in Figure 5. In most of the designs, multistate design chose the native Ral amino acid for positions which are important for Ral stability, or for RalBP1 binding. For example, serine-50, which is consistently recovered, forms hydrogen bonds with two residues on RalBP1, threonine-437 and glutamine-433. Similarly, tyrosine-82, in the core of the interface, maintains its contact with RalBP1 histidine-413. The wild type leucine at the very buried position 67 is the most frequently selected amino acid at that position. Tyrosine is also designed frequently at this position because it can form a good intramolecular hydrogen bond with arginine-78. Similarly, multistate design preferred arginine or histidine, instead of the wild-type lysine, at position 16 because of weak intramolecular hydrogen bonds.

Multistate design readily identified positions that destabilized the negative states. Ral positions 36 and 52 are important specificity positions for Sec5. Multistate design favored lysine at position 36 because it creates a clash with Sec5 residue glycine-28. Similarly, it liked to mutate position 52, an arginine in wild-type Ral which points out into solvent, to phenylalanine, leucine, or isoleucine. These residues all create clashes with threonine-28 on Sec5. Several positions appear to be important for preventing association with Exo84. Multistate design frequently mutated residue 14 to glutamic acid which clashes with a loop in Exo84. The wild type asparagine at position 81 in Ral makes two sidechain-backbone hydrogen bonds with Exo84. Multistate design changed this position to aspartic acid exclusively, and its sidechain cannot form hydrogen bonds in the low-energy redocked Exo84 structures. This aspartic acid also interacts favorably with RalBP1's lysine at residue 421 in the Ral/RalBP1 design models. Any large, bulky residue at position 78 can produce a clash with Exo84. Multistate design favored placing arginine at this position, but even leucine is able to destabilize this interface. Finally, multistate design almost always placed either the wild-type alanine or a serine at position 77. Serine is a good choice for this position as it forms a small clash with the Exo84 backbone and adds a favorable interaction with RalBP1 residue glutamine-417.

A number of positions, specifically 47, 73, 74 and 75, displayed no clear preference among the designed sequences. Multistate design generally favored placing polar amino acids at these positions given that they are surface-exposed. Except for position 47, none of these positions look like they could provide significant amounts of specificity to the interface. The wild type Ral tyrosine at position 75 participates in a hydrogen bond with Exo84 serine-276. Multistate design removed this favorable interaction, and placed mostly histidine and arginine at this position. Positions 63 and 65, natively glutamine and aspartic acid, respectively, are in the middle of a beta-sheet in RalA and were also mutated to a wide variety of amino acids. Multistate design displayed a small preference for glutamic and aspartic acids at these positions, respectively. These mutations make sense as in the wild-type Ral/RalBP1 structure an arginine residue on RalBP1, arginine-434, interacts with the aspartic acid at Ral position 65. This same arginine residue can interact with a glutamic acid at position 63, if an aspartic acid at position 65 is not present.

## Discussion

Here we have presented a generic implementation of multistate design which allows users to rapidly customize the fitness function to be optimized, and have shown how the implementation can be used in two related, yet distinct, design tasks. In fact, the ease with which new states can be added and their energies managed through the fitness-function-definition file made it possible to perform multiple rounds of negative design with increasing diversity in the conformations available to the negative states, thereby increasing multistate design's accuracy, which to our knowledge has not previously been reported.

**Figure 6:**
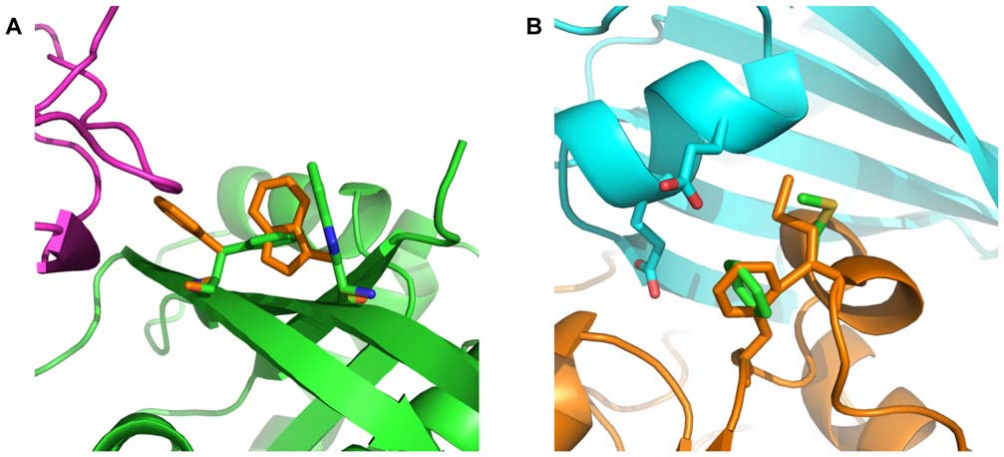
Curious cases from negative design. A) Placing both F52 and W63 on RalA (green) destabilizes its interaction with Sec5 (magenta). In the docked conformation, the F52 and W63 rotamers collide in the least-awful-rotamer placement available. In the unbound state (orange) these residues relax out of collision. W63 disrupts binding with Sec5 through F52, but neither residue disrupts binding on its own. Unfortunately, W63 is incompatible with the RalA backbones from the crystal structures, though it is compatible with the RalB backbone in the NMR structure. Here, a discrepancy between the backbone conformations of Ral in its various states lead to a questionable design. B) The Missing Rotamer Problem encountered while trying to redesign chain E of human uracil-DNA glycosylase bound to a protein inhibitor (PDB ID: 1UGH). The mutation F267 on chain E (green) is consistently chosen by multistate design when optimizing for binding energy, not because F267 forms favorable contacts with its (polar) neighbors on chain I (cyan), but because the rotamer it finds in the bound state is absent from the set of rotamers for the unbound state; the best available rotamer for the unbound state (orange) has a high-energy collision with the C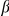 atom on residue 279. The green rotamer collides with the chain E backbone (with an energy 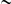
5.1 REU) and, in the unbound, is pruned by Rosetta's bump check machinery (threshold of 5.0 REU); however, in the bound state, slightly favorable interactions with the chain I backbone rescue this rotamer by pushing its energy just beneath the bump-check threshold (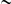
4.9 REU). Placing phenylalanine at 267 and anything besides glycine at 279 produces a large energy difference in the bound and unbound states which masquerades as an excellent binding energy.

The implementation separates its search through sequence space and conformation space as many prior examples of multistate design have [26], [27], [29], [52], as opposed to their simultaneous optimization in the belief-propagation algorithm presented by Fromer *et al.* [53], or the reduced-representation, sequence-space-only optimizations presented by Nautiyal *et al.* [54] and by Grigoryan *et al.* [28], [55]. The explicit rotamer optimization we perform in our inner loop was able to find interesting through-residue interactions where one residue can pre-order a neighboring residue such that this second residue's interaction with a third residue would be unfavorable (Figure 6A); in contrast, Grigoryan *et al.* 's [55] score function, which represents amino-acid pair interactions by their average rotamer-pair-interaction energies, would be unable to capture the pre-ordering effect that the first residue exerted on the second.

In contrast to the multi-specificity algorithms presented by Humphris and Kortemme [52] and Fromer *et al.* [53], [56], the implementation is suited to perform both positive and negative design. We have tuned the parameters of our genetic algorithm to behave as well as Rosetta's existing single-state design algorithm at single-state design problems, but we have not compared the genetic algorithm's performance to the intriguing FASTER-MSD algorithm presented by Allen and Mayo [57], whose implementation starting from our existing code should be straight forward.

Our design protocol does not explicitly consider side chain entropy, as the energy of a sequence threaded onto a backbone is calculated by searching for the combination of rotamers with the lowest energy. In contrast, in their minDEE/K* algorithm Georgiwev, Lillen and Donald [58] return a distribution of energies for alternative side chain packing arrangements of a single sequence. Such distributions may be useful, as they could capture the sidechain entropy of unbound conformations, though the role sidechain entropy plays in protein structure remains unclear [59], [60].

It turns out that there are many more pitfalls of performing negative design than of performing positive design. For our implementation to be robust to any possible fitness function, where any state could end up playing both a negative and a positive design role, we had to ferret out the several ways in which multistate design can fail. In the remainder of this section, we present our insights into these challenges as they might prove useful for other researchers interested in performing negative design. These challenges derive from three problems: suboptimal rotamer placement, the missing rotamer problem, and the fixed-backbone assumption.

### Suboptimal rotamer placement

**Figure 7:**
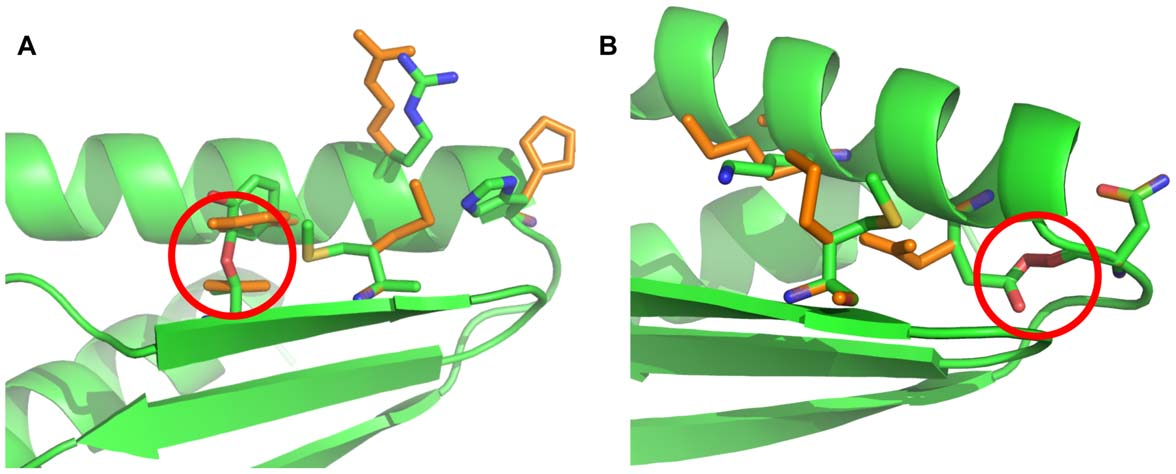
Packing failures from the (negative state) monomers of 1USM. A) Due to the nature of the BMEC+sPR algorithm, it systematically failed to relieve the Y28/T44 collision in the presence of M46. The colliding rotamers are shown in green; the collision-free placement is shown in orange. The collision is highlighted with a red circle. When multistate design encountered these three amino acids, the bound state produced a decent energy, the unbound state produced a high energy, and the strength of the apparent binding energy caused the fitness to be exceptional. Since this is a systematic failure, all the sequences in the genetic algorithm's pool at the end of the design trajectory that produced this sequence contained these three amino acids and their unrelieved collision. Fortunately, not all multistate design trajectories encountered these three amino acids together. B) The Multicool Annealer also fails randomly; in one multistate-design trajectory, a single packing failure left an unresolved collision (red circle) between E24 and the backbone of D20 (green sidechains) that was in fact resolvable (orange sidechains). This collision made this sequence appear to have the best fitness. Since this was a random and not a systematic failure, none of the other sequences in the genetic algorithm's pool exhibited this flawed packing.

If, when optimizing the rotamers for a negative state, the optimization algorithm should fail to find the optimal energy (*e.g.* by leaving a collision between two rotamers), then the calculated fitness will be better than what it would be if the optimization algorithm had succeeded; the larger the failure, the better the computed fitness will be. There are two ways that packing algorithms can fail: systematically and randomly (Figure 7). Both are problematic, but systematic failure – where a portion of a sequence is consistently mispacked – corrupts the entire population of sequences (Figure 7A). That is, if a portion of a sequence leads to a systematic packing failure, then point mutants of that sequence will also lead to systematic packing failures and will also have very favorable fitnesses. Eventually the pool of sequences the genetic algorithm keeps will fill entirely with those sequences which produce the systematic packing failure. None of the designs produced by such a trajectory are worth examining. On the other hand, random failure, where as a rare event a collision remains unresolved in a negative state, will not result in the corruption of the entire pool of sequences. The neighboring sequences to the one which produced the packing failure will not be any more prone to packing failures than any other, so the pool of sequences the genetic algorithm keeps will not fill up with sequences that produce packing failures (Figure 7B).

Since packing failures are likely to occur in any given multistate design trajectory, it is important to select a fitness function that avoids overly rewarding mispacked sequences. In the first fitness function used in the heterodimerization task, packing failures of the monomers produced large apparent binding energies which in turn were seen as very favorable. That is, once the homodimeric states were fully destabilized, the fitness function simplifies to: 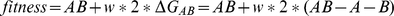. Here, because the monomers' energies sit behind a minus sign, they are negative states; their destabilization would improve the fitness. However, in this task, there is no driving motivation to destabilize the monomers; in fact, destabilizing the monomers to the point of their unfolding would be highly undesired. To address this problem, we tested a second fitness function to limit the reward for improving the heterodimer binding energy. This fitness function capped the reward for the heterodimer binding energy once it reached −24 REU; effectively, the monomers were negative states only if the binding energy was less than −24 REU, and were no longer negative states once the binding energy crossed that threshold.

### The missing rotamer problem

If a rotamer is present in a positive state (e.g. the bound conformation of two chains, A and B) and absent in a corresponding negative state (e.g. the unbound conformation of chain A), multistate design will exploit its absence producing designs of dubious quality (Figure 6B). Just as in the suboptimal-rotamer placement case, if a negative state (but not its corresponding positive state) is assigned a high energy, then the computed fitness will be better than it should be. Absent rotamers allow this opportunity: the “good rotamer” that's needed in the negative state is absent and is chosen in the positive state. If the rotamer had not been absent, then the sequences leveraging the missing rotamer would not have produced favorable fitnesses. Missing rotamers effectively create systematic packing failures.

Rosetta's logic for building rotamers has three opportunities to build different rotamers for different states. First. Neighbor-dependent extra-rotamer building logic: by default, the addition of extra rotamers by taking samples at 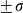 for 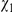 and 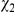 through the use of the “-ex1” and “-ex2” flags is only performed at residues with more than 18 C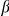 neighbors within 10 Å. Residues in unbound states often have fewer neighbors than the same residues in the bound states, so the default behavior would generally build fewer rotamers in the (negative) unbound states than in the (positive) bound states. To avoid this problem, this default behavior is disabled in multistate design so that extra rotamers are built at all residues. As a consequence, multistate design has to consider many more rotamers than does single state design.

Second. Backbone-collision filter to remove rotamers (*bump check* in Rosetta jargon): Rosetta calculates the Lennard-Jones energy for a rotamer with the background and discards rotamers whose energies exceed a threshold (as is commonly done). We have encountered a case where a phenylalanine rotamer on one chain in the unbound state collides with its backbone slightly beyond Rosetta's default threshold for exclusion, but is rescued in the bound state by favorable contacts across the interface (Figure 6B). When multistate design optimized the binding energy across this interface, it always selected phenylalanine at this residue, in spite of high energy in the bound state, because the energy in the unbound state was dramatically worse and the apparent binding energy was excellent. Unfortunately, the solution to this problem would not have been to simply use the repulsive component of the Lennard-Jones term in the bump-check scoring and to exclude the attractive component which, here, rescued the high-energy rotamer. If the tables had been turned so that the dimer was the negative state, the monomer the positive state, and bump check pruned the PHE rotamer for the dimer but not the monomer, then exactly the same situation would have arisen where a missing rotamer would have produced an apparent, but incorrect, destabilization of the dimer over the monomer. Our solution to this problem was to disable the bump check filter. As a consequence, multistate design has to consider many more rotamers than does single state design.

Third. Backbone-dependent rotamer building: Rosetta uses the 2002 version of Dunbrack's backbone dependent rotamer library [61]. This means that the set of rotamers built for one backbone might not be the same set of rotamers built on another backbone. This problem unfortunately changes the “fixed backbone assumption” of multistate design into a somewhat less desirable “fixed backbone requirement.” In light of this problem, we restricted our simulations to only compare energies between states with the same backbone conformations. In our docking trajectories, we similarly performed rigid-body docking only to prevent alternate backbones from being added into the negative states in subsequent rounds. The missing rotamer problem could be avoided entirely if, instead of performing discrete rotamer optimization, we performed continuous rotamer optimization [62], [63], but this would surely come at the price of longer running times since rotamer-pair energies could not be saved and reused.

*Fixed backbone assumption*. There are three ways in which the fixed-backbone assumption affected our results. The first two relate to the fixed-backbone assumption's restrictions on the rigid-body degrees of freedom connecting the two chains, the third relates to the restriction on the internal degrees of freedom in each individual chain. First, we found in preliminary trajectories, before we introduced the cap at 0 for the binding energies of the undesired interactions, that multistate design would often introduce the largest collision it could into the negative states in order to increase the gap between the positive- and negative-state energies. Allen and Mayo observed a similar behavior in negative design and chose to cap repulsive interactions between residue pairs at +50 energy units [57]. In the heterodimerization task, multistate design often introduced collisions into one of the two homodimers while failing to destabilize the other homodimer. The fitness function rewarded a pair of binding energy gaps of (−1000 REU, +3 REU) more than it rewarded binding energy gaps of (−10 REU, −10 REU) even though a binding energy gap of −1000 REU was physically impossible. This problem is due to the fixed backbone assumption. Since the backbones are held fixed, they cannot separate to remove strain across the interface. There is a subtle issue here: once the apparent binding energy from a particular conformation goes positive, that conformation can no longer be considered valid. Binding energies cannot be positive. Instead, the model of two proteins held rigidly docked against each other breaks down. There are two solutions to this problem: use a cap in the fitness function to limit the negative-state binding energies at zero (which we did) or add an alternate undocked conformation containing both chains, but where the chains are physically separated by 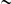
20 Å; this “undocked” conformation for the dimer would presumably be chosen as the minimum energy conformation once collisions had been introduced into all the other docked conformations. The advantages of the first solution are that it avoids any errors stemming from packing discrepancies between the unbound monomers and the undocked dimer, and that it is one state (one CPU) per negative species cheaper to execute. The use of a cap on the binding energies for the negative states is original to this study and has the clear advantage of focusing multistate design's efforts on stabilizing the positive states once the negative states have been fully destabilized.

Second, we found that rigid-body docking was often able to relax away collisions present in the homodimeric sequences that came out of the early rounds of design. Multistate design can only design against states it can see, and there are a surprising number of low-energy docked conformations for our homodimers. Keating *et al.* [64] similarly noticed that allowing their backbones to relax after introducing mutations improved their ability to predict the adopted conformations and binding energies of their hetordimeric coiled-coils. Havrakek and Harbury [26] noticed that a single round of multistate design overstated the destabilization of the heterodimeric species they were designing against; they suggested that the addition of more states could overcome this problem and our *in silico* results are consistent with this hypothesis.

The third way that the fixed backbone assumption impacted our results is more difficult to describe. In the setup-scheme 1 designs for the RalA task, multistate design found a pair of mutations, W63 and F52 (Figure 6A), where the binding with Sec5 was disrupted, but at the cost of destabilizing the RalA backbones taken from the crystal structures (states 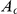 and 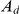). In contrast, the NMR models of RalB bound to RalBP1 were able to accommodate these mutations. Since the 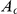 and 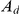 energies of the RalA monomers from the negative states were invisible to the fitness function, multistate design dutifully chose these mutations. The destabilization of the backbone conformation for RalA from the Sec5 crystal structure is worrisome in this case because the section of the RalA backbone being designed has such high agreement between the Sec5-bound and Exo84-bound crystal structures (though, the RalBP1-bound NMR models showed significant disagreement). We did not want to disrupt the crystal conformation. The fixed-backbone assumption was more of a requirement in this case: we designed for a backbone we were unsure about (the NMR model) without considering a backbone we were interested in preserving (the crystal backbone), but, if the same backbone had been present in all three models, we would not have encountered this issue. We tried twice to skirt this problem by docking the crystal structure of RalA against the RalBP1 models, and by docking the RalB-NMR structures against the Sec5 and Exo84 models, but neither approach resulted in good docking funnels or satisfactory binding energies.

There were two possible solutions to this problem: to modify the fitness function to disfavor the destabilization of the RalA crystal structure, or to redefine the set of positions which are allowed to design. Taking the first approach, one could have included the energies of the crystal forms of the unbound RalA states in the fitness function: 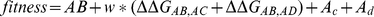. Such a fitness function has the unfortunate consequence of triple-counting stabilizing mutations to the RalA structure. Alternatively, one could penalize the destabilization of the crystal forms of RalA beyond some threshold: 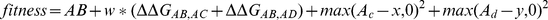 where 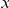 and 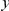 are some predetermined constants representing an upper bound on how destabilized the RalA monomers could become before the penalty kicks in. We went with the second option and expanded the set of designable positions. This had the serendipitous effect of favoring sequences on the RalA backbone which were compatible with all three structures; the fitnesses for the best designs which lacked the F52/W63 pair were better than those with them.

## Supporting Information

File S1 “Protocol capture” file that contains the input files, command lines, and support scripts that were used in this computational study.
(BZ2)

File S2 Design models generated by this study for both the heterodimerization task and the orthogonal interface redesign task.
(BZ2)
